# Significance of NPM1 Gene Mutations in AML

**DOI:** 10.3390/ijms221810040

**Published:** 2021-09-17

**Authors:** Andrew Hindley, Mark Alexander Catherwood, Mary Frances McMullin, Ken I. Mills

**Affiliations:** 1 Clinical Haematology, Belfast City Hospital, Belfast BT9 7AB, UK; mark.catherwood@belfasttrust.hscni.net; 2 Centre for Medical Education, Queen’s University Belfast, Belfast BT7 1NN, UK; maryfrances.mcmullin@belfasttrust.hscni.net; 3 Northern Ireland and Belfast Health and Social Care Trust, Belfast BT9 7AB, UK; 4 Patrick G Johnston Center for Cancer Research, Queens University Belfast, Belfast BT9 7AE, UK; k.mills@qub.ac.uk

**Keywords:** AML, *NPM1*, fragment analysis, *FLT3*, *DNMT3A*

## Abstract

The aim of this literature review is to examine the significance of the nucleophosmin 1 (*NPM1*) gene in acute myeloid leukaemia (AML). This will include analysis of the structure and normal cellular function of NPM1, the type of mutations commonly witnessed in *NPM1*, and the mechanism by which this influences the development and progression of AML. The importance of *NPM1* mutation on prognosis and the treatment options available to patients will also be reviewed along with current guidelines recommending the rapid return of *NPM1* mutational screening results and the importance of employing a suitable laboratory assay to achieve this. Finally, future developments in the field including research into new therapies targeting *NPM1* mutated AML are considered.

## 1. Introduction

Acute myeloid leukaemia (AML) is a rare haematological disorder characterised by excess proliferation of haematopoietic precursors of myeloid lineage. Primarily originating in the bone marrow, disease progression is often characterised by infiltration into the peripheral blood system and other tissues. Whilst presenting at all ages, incidences of AML increase with age with the median age at diagnosis in the UK being 70.6 years [1]. In Northern Ireland, the rate of incidence is 3.8 per 100,000 population with 33 new cases reported in 2017 [2]. Despite differing significantly based on age, 5-year overall survival rates for AML remain disappointingly low at approximately 20%, with AML accounting for less than 1% of cancer diagnoses but approximately 2% of all cancer deaths in the UK [3]. Advances in molecular biology have led to significant insights into the heterogeneity of AML down to the genetic level, with this knowledge providing valuable information on classification, prognosis and treatment options for individuals depending on the genetic mutations present.

## 2. Classification of Acute Myeloid Leukaemia

Leukaemia as a pathological disease was beginning to be discussed in the literature in the mid-19th century following observation of excess white blood cells in patients post-mortem with the term ‘leukaemia’ coined in 1847 [4,5,6,7,8]. In the 1970s the French-American-British (FAB) classification system subdivided into seven categories (M0–M7) differing in stage of maturation and the myeloid lineage of the proliferating clone [9]. From the mid-20th century, advancements were made in genetic sequencing allowing genetic mutations to be studied and cytogenetic techniques such as karyotyping and fluorescent in situ hybridisation allowed identification of chromosomal aberrations [10,11]. Immunophenotyping by flow cytometry has also become an important tool in classification by allowing the pattern of surface antigens on leukaemic cells to be determined using monoclonal antibodies. 

The utilisation of such techniques in the study of haematological malignancies has highlighted the diverse nature of the disease at the genetic, cytogenetic and immunophenotypic level and led to a major reclassification by the World Health Organisation (WHO) in 2001, which accounted for recurrent genetic abnormalities known to be of significance in AML [12]. Further advancements in knowledge required revisions in 2008 and 2016 [13,14]. The 2016 classification of AML with recurrent genetic abnormalities (Table 1) primarily consists of chromosomal translocations with only two single-gene mutations included, biallelic mutation in the transcription factor gene CCAAT/enhancer binding protein alpha (*CEBPA*) and AML with a mutated nucleophosmin 1 (*NPM1*) gene [15]. Both single gene mutations were included as unique classifications having been provisional entities in the 2008 classifications. 

## 3. Nucleophosmin 1 Structure

**Figure 1:**
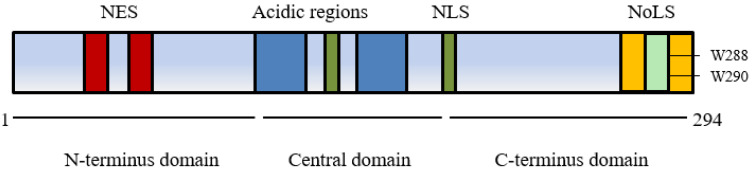
Nucleophosmin 1 protein structure showing the nuclear export signal (NES) in the N-terminus domain, the nuclear localisation signal (NLS) of the central domain and the nucleolar localisation signal (NoLS) in the C-terminus domain containing tryptophan at amino acid positions 288 and 290 [17].

The nucleophosmin 1 gene is located on chromosome 5q35 and consists of 12 exons [16]. It encodes a multifunctional protein comprised of 294 amino acids (35–40 kDa). The structure of *NPM1* and the presence of several nuclear import and export signals contribute to its shifting cellular localisation and associated functions (Figure 1) [17]. 

The conserved N-terminus of *NPM1* has two nuclear exporter signals (NES) that facilitate movement from the nucleoplasm to cytoplasm in association with a nuclear exporter protein exportin 1/chromosomal region maintenance 1 XPO1/CRM1 and has a significant role in binding a wide range of proteins [18]. Post translational modification also influences cellular movement via self-oligomerisation of NPM1 to pentameric form promoting export from the nucleolus with phosphorylation controlling monomerization and a return to nucleolar localisation [16,19]. Within the central domain two nuclear localisation signals (NLS) lead to movement from cytoplasm to nucleoplasm whilst a C-terminus domain with two nucleolar localisation signals (NoLS) containing highly conserved tryptophans at positions 288 and 290 plays a crucial role in localising NPM1 back to the nucleolus [20]. 

## 4. Nucleophosmin 1 Function

Numerous functions of NPM1 have been described and are often facilitated by the ability to shuttle intracellularly between nucleoli, nucleoplasm, and cytoplasm.

### 4.1. Ribosome Biogenesis

NPM1 plays a role in cell growth and proliferation by participating in ribosomal biogenesis in the nucleolus which is essential for protein translation. NPM1 has been shown to interact with ribosomal protein L5 facilitating export from the nucleolus of the larger ribosomal subunit 60 S containing ribosomal RNA (rRNA). The blocking of rRNA export via NPM1 inhibition has been shown to lead to cell cycle arrest [21]. 

### 4.2. Histone Chaperoning

NPM1 is an important histone chaperone necessary for the organisation of chromatin as well as assisting in chromatin transcription. The association of NPM1 to histone proteins is thought to be due to an abundance of negatively charged amino acids in the acidic region which mimic the ionic bonding of histones with DNA/RNA [22]. NPM1 chaperones the histone linker protein H1 to chromatin resulting in the reversible binding of H1 to chromatin which facilitates a change to the more conformationally condensed heterochromatin [23]. Additionally, NPM1 promotes chromatin transcription via interaction with the core histone proteins H2B, H3 and H4 following acetylation [24].

### 4.3. Centrosome Duplication

NPM1 is closely associated with centrosome activity and helping to maintain faithful cell division. NPM1 associates with unduplicated centrosomes via its nuclear export signal [25]. Centrosomal disassociation of NPM1 via phosphorylation by CDK2/cyclin E allows centrosome duplication to take place [26]. Knockout mice completely lacking the *NPM1* ortholog *Npm1* gene have been shown to have uncontrolled centromere duplication and are not viable embryonically past day 16 due to impaired organogenesis [27].

### 4.4. Nucleolar Structure

Despite being a membrane-less organelle, the nucleolus is organised into three sub compartments: a fibrillar centre (FC) surrounded by a dense fibrillar component (DFC) which is encompassed by the granular component (GC) [28]. The GC is mostly comprised of pentameric NPM1 that separates from the fibrillin rich DFC via liquid–liquid phase separation, which separates the proteins in a similar fashion as oil, and water [29]. The protein layers separate based on their surface tension and the most energy efficient conformation. As NPM1 has the lowest surface tension present, it naturally is ordered on the outer surface of the nucleolus. The layered protein rich structures of the nucleolus are believed to be important for the directionality of ribosome biogenesis proceeding from the inner to outer nucleolus [30].

### 4.5. DNA Repair and Tumour Suppression 

NPM1 plays an important role in DNA repair, helping to modulate apurinic/apyrimidinic endonuclease 1 (APE1) protein activity. APE1 plays a crucial role in the base excision repair (BER) pathway. NPM1 facilitates its function by influencing localisation in the nucleoli and promoting endonuclease activity [31]. NPM1 directly influences the activity of the tumour suppressor protein TP53 in response to cellular stress such as that caused by UV damage and RNA polymerase 1 inhibition [32]. TP53 activity is negatively controlled by interaction with murine double minute 2 (MDM2) protein in the nucleoplasm. Upon cellular stress, NPM1 moves from the nucleolus to the nucleoplasm where it binds MDM2, inhibiting MDM2 mediated degradation of TP53, leading to an increase in TP53 activity which results in cell cycle arrest or apoptosis. 

Due to such wide ranging and important cellular functions, it is not surprising that aberrant functioning of NPM1 has been found in a wide range of solid tumours such as prostate, ovarian, gastric and colon [33].

## 5. Mutations in Nucleophosmin 1 in AML

*NPM1* is the most commonly mutated gene in adult AML, present in approximately 25–35% of patients [34]. It is less frequent in children with approximately 8% of childhood AML patients harbouring an *NPM1* mutation [35]. *NPM1* mutated AML often presents with a high white blood cell and blast count with *NPM1* mutations observed in all AML subtypes under the FAB classification system except acute promyelocytic leukaemia [36]. *NPM1*-mutated AML typically has cytological characteristics of either myeloblastic leukaemia with/without differentiation or acute leukaemia with monocytic/monoblastic differentiation and cup-like nuclei often being observed in blast cells [37]. Immunophenotypically, *NPM1* mutated cells are often positive for the surface antigens CD33, CD117 and MPO in myeloblastic like cases, whilst CD64 and CD14 positivity is typical in cells with monocytic differentiation. Over two thirds of cases have blasts that are CD34 negative whilst a third are negative for HLA-DR [38]. The immunophenotypic pattern along with morphological features may be suggestive of *NPM1*-mutated AML early in laboratory investigations.

The *NPM1* locus is involved in translocations causing hematologic malignancies such as acute promyelocytic leukaemia where t(5;17)(q35;q12) leads to *NPM1-RARα*, anaplastic large cell lymphoma where t(2;5)(p23;q35) leads to *NPM1-ALK*, and myeloid neoplasms where t(3;5)(q25;q35) leads to *NPM1-MLF1* [39,40,41]. Recently, three new NPM1 translocations were identified in cases of AML: (i) t(5;10)(q35;q23); (ii) t(5;18)(q34;q12), and (iii) t(5;6)(q35;q23), generating the NPM1/RPP30, NPM1/SETBP1 and NPM1/CCDC28A fusion transcripts [42].

Initial investigations into the role of *NPM1* mutations in AML followed on from the study of the chimeric fusion protein NPM-ALK which is present in 30–50% of advanced anaplastic large-cell lymphoma cases [43]. Immunohistochemical staining showed that in these cases NPM1 localised to the cytoplasm as opposed to the nucleolus as seen in normal tissue [44]. An expansion of testing into other types of leukaemia led to a trial enrolling 591 AML patients in 1999 with Falini and colleagues demonstrating a high proportion of cases showed cytoplasmic localisation of NPM1 in AML, the primary cause of which was identified as mutations in exon 12 of *NPM1* [45]. 

### 5.1. NPM1 Mutations

**Figure 2:**
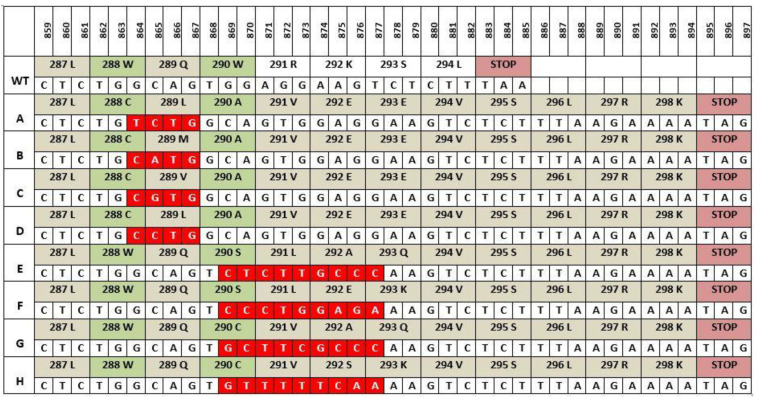
NPM1 exon 12 mutations. Mutation types (A-H) differ in the composition and number of nucleotides inserted (shown in red). All mutants shown have the same 4 amino acid addition at the end and an alteration to at least one of the tryptophans at positions 288 and 290 [42].

The majority of *NPM1* mutations are found in exon 12 and approximately 50 different *NPM1* mutation types have been described with the most common being Type A, which involves a TCTG tetranucleotide duplication and accounts for 75–80% of all *NPM1* mutations in AML [46]. The next most common mutation, type B accounts for 10% of all mutations and involves a 4-base pair (bp) insertion (CATG) at the same nucleotide position as type A. Type D, the third most common mutation possesses a similar 4bp insertion of CCTG and is present in 5% of *NPM1* mutations (Figure 2). Rare NPM1 mutation have also been described in exons 5, 9 and 11 which also cause aberrant cytoplasmic NPM1 localization [42].

Recent research has suggested a mechanism involving terminal deoxynucleotidyl transferase (TdT) as being responsible for the duplication witnessed in *NPM1* mutations [47]. TdT is a unique polymerase whose main function is to add non templated nucleotides during V (D) J recombination in early B lymphoid cells with a preference for GC additions contributing to antigen receptor diversity and playing a crucial role in adaptive immunity [48,49]. Borrow and colleagues have shown that replication slippages by TdT may be responsible for *NPM1* mutations in AML and that the difference in the proportion of *NPM1* mutations in AML between children and adults may arise from higher TdT activity in children. The eight types of mutations shown in Figure 2 all differ in position of insertion and the nucleotide composition, yet all share several similarities. All mutations result in a frameshift mutation leading to an extension of NPM1 by four amino acids (aa) from 294aa to 298aa and, most importantly, every mutation results in a change to at least one of the tryptophan amino acids at position 288 or 290.

### 5.2. Cytoplasmic Relocation of Mutated NPM1

The different mutations mentioned result in the same defining feature of mutated NPM1, permanent cytoplasmic localisation which appears to be the driving force for leukemogenesis. Losing one or more tryptophan residues at the C-terminal leads to a loss of the NoLS whilst simultaneously gaining a leucine rich NES. Loss of tryptophan at 290 alone results in a stronger NES than the loss at positions 288 and 290 [50]. Export of mutated NPM1 is facilitated by XPO1 which has a greater affinity for the mutant C-terminal NES than the wild type N-terminal NES leading to cytoplasmic accumulation [51].

In *NPM1* mutated AML the loss of all NPM1 nucleolar localisation is perhaps surprising as *NPM1* mutations are always heterozygous in nature due to the non-viability of homozygous *NPM1* mutations. The localisation of wild type NPM1 in the cytoplasm is believed to be due to the formation of heterodimers of NPM1wt/NPM1mut which are relocated to the cytoplasm [17].

This raises the question of what is the mechanism by which cytoplasmic localisation triggers the leukaemic state in *NPM1* mutated myeloid cells? One set of genes known to be highly upregulated in AML are the class I homeobox (*HOX*) genes which play a crucial role in proliferation, differentiation, and self-renewal in haematopoietic stem cells [52]. It is believed that an upregulation in *HOXA* and *HOXB* genes leads to a stem-cell like state [53]. Recent research has shown that NPM1mut influences upregulation of *HOXA* and *HOXB* genes as witnessed by epigenetic modification of Histone 3 (H3). H3 is acetylated at leucine position 27 (H3K27ac) to enhance transcription of *HOX* genes in NPM1mut AML [54]. To prove that NPM1mut directly causes HOXA and HOXB overexpression as opposed to it being a consequence of stalled differentiation, Brunetti and colleagues used CRISPR-Cas9 gene editing to interrupt the NES in NPM1 mut resulting in nuclear re-localisation and subsequent differentiation and arrest in AML cells, showing that *HOX* gene overexpression is facilitated by NPM1mut. The exact mechanism by which NPM1mut upregulates HOX expression is not yet known, although research has pointed to involvement of the histone methyltransferase mixed lineage leukaemia 1 (MLL1) in association with its cofactor menin in the upregulation of Meis homeobox 1 (MEIS1). Transcriptionally active MEIS1 is believed to be a cofactor in the expression of *HOX* genes in early leukaemic development in AML and NPM1mut AML is believed to be dependent on the MLL1-menin interaction for increased *HOX* gene expression [55,56].

Due to the wide range of functions of NPM1wt it has also been suggested that NPM1mut might also contribute to leukaemic phenotype by chaperoning other molecules to the cytoplasm, altering their function. The N-terminal of NPM1mut appears to be unaffected in its interactions with other molecules, associating with them as normal and then transporting them to the cytoplasm. The irregular cytoplasmic localisation of associated proteins involved in DNA repair pathways, apoptosis, and differentiation such as alternate reading frame (ARF), apurinic/apyrimidinic endonuclease 1 (APE1) and mouse double minute 2 (MDM2) may be involved in NPM1mut leukaemia, but there is not yet any definite proof that this plays a significant role.

It had been reported that the transcriptional factor PU.1/spi-1 responsible for monocytic-granulocytic differentiation is translocated to the cytoplasm in association with NPM1mut and possibly contributes to arrest of differentiation, conceivably resulting in the monocytic features common in NPM1mut AML [57]. Recent research has queried the level of cytoplasmic localisation of NPM1mut -PU.1 [58], the results of which are still being debated [59].

A similar effect of cytoplasmic relocation altering function has been reported in the interaction of NPM1mut with bromo-domain protein 4 (BRD4) which is an epigenetic regulator of transcription. NPM1wt inhibits BRD4 in the nucleus and cytoplasmic relocation of NPM1 leads to a reduction in this inhibition with BRD4 increasing transcription in its target genes, the anti-apoptotic *bcl-2* and oncogene *c-MYC* [60].

## 6. Coexisting Mutations in *NPM1*mut AML

Like most cancers, AML does not result from a single gene mutation and usually requires the sequential acquisition of numerous genetic mutations in a single lineage over time for disease evolution. One of the precursors to AML is clonal haematopoiesis, where a number of somatic mutations exist in haematopoietic cells without evidence of malignancy [61]. *NPM1* mutations occurring in a cell population with evidence of clonal haematopoiesis appears to be sufficient to progress the clone to development of AML. The absence of *NPM1* mutations in cases of clonal haematopoiesis is further evidence that NPM1 is a ‘driver’ or ‘gateway’ mutation needed for progression to AML in NPM1mut cases.

**Figure 3:**
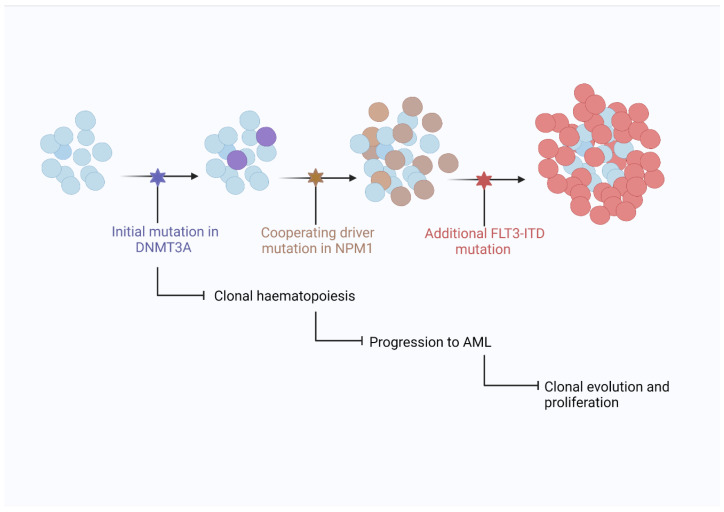
Model showing progression of clonal haematopoiesis to AML. Clonal haematopoiesis is believed to exist prior to AML development with mutations in *DNMT3A* often present. The acquisition of an *NPM1* mutation is considered a driver mutation resulting in transformation to AML with further mutations in *FLT3-ITD* driving proliferation and establishing a predominant clone [20].

Many genes with diverse roles in cellular activities such as signalling, epigenetic modulation, chromatin modification, RNA splicing, and tumour suppression are commonly mutated in AML [34]. Patterns of common, co-existing gene mutations have been observed in AML and helped define a model explaining clonal haematopoiesis’ progression to AML. Figure 3 shows an example of disease progression in NPM1mut AML and the closely associated mutations in DNA methyltransferase 3 alpha (*DNMT3A*) and FMS like tyrosine kinase 3 (*FLT3*) genes.

### 6.1. DNMT3A

DNA methyltransferase 3 alpha (DNMT3A) is a protein responsible for DNA methylation, mutations in which are found in approximately 20% of all AML cases and as a co-mutation with NPM1 in 50% of cases [62,63]. *DNMT3A* appears to be a preleukaemic mutation commonly existing in a clonal population before additional mutations drive the development of AML. *DNMT3A* is often still present in remission indicating *DNMT3A* mutations are not a key driver of AML development [64].

### 6.2. FLT3

The FMS like tyrosine kinase 3 (*FLT3*) gene encodes a transmembrane receptor tyrosine kinase normally expressed in haematopoietic stem cells that contributes to signalling in P13K, RAS and JAK-STAT pathways [65]. Mutations via internal tandem duplication (ITD) or in the tyrosine kinase domain (TKD) lead to aberrant cell proliferation due to constitutively active FLT3 kinase and are present in around 25% of AML cases, with almost a third of NPM1 mutations occurring in association with a *FLT3* mutation [66]. FLT3 has been a therapeutic target in recent years with several tyrosine kinase inhibitors (TKI) including midostaurin and second generation TKIs such as gilteritinib being licenced for use in patients with *FLT3* mutated AML. *NPM1*, *DNMT3A* and *FLT3* are the three most mutated genes in AML with some research showing them as the most commonly occurring three gene co-mutations, present in up to 6% of patients and conferring a poor prognosis as has also been demonstrated in murine studies [67,68]. This highlights the complexity of the genomic mutational landscape even within individuals and the extent to which the pattern of mutations and the presence or absence of multiple mutations in relation to each other can affect disease course and prognosis.

## 7. Prognostic Significance of *NPM1* Mutation in AML

Increasing knowledge of co-mutations and particularly the relationship of *NPM1* mutations with those in the *FLT3* gene has given clinicians important prognostic information and helped guide treatment decisions. The European Leukaemia Network (ELN) guidelines released in 2017 stratified risk into the categories of favourable, intermediate, and adverse depending on the genetic abnormalities present (Table 2) [69].

*NPM1* mutational status helps determine prognosis but is dependent on the presence or absence of a *FLT3* mutation and its allelic ratio. Interestingly, the presence of an *NPM1* mutation particularly in the absence of a *FLT3* mutation and with a normal karyotype confers a favourable prognosis. The reason for this favourable outcome and response to treatment is not entirely clear; in recent years, research has suggested that NPM1mut may interact directly with nuclear factor-κB to improve sensitivity to standard chemotherapy treatment [70]. NF-κB is a protein involved in cell growth and proliferation, inflammation, anti-apoptosis and is constitutively activated in AML cells and plays a key role in chemoresistance in numerous malignancies including AML [71,72]. Zhang and colleagues showed that in vitro, type A NPM1 mutation reduced transcriptional activity of NF-κB and increased apoptosis in cells treated with the chemotherapy drugs daunorubicin and cytarabine and reduced expression of the anti-apoptotic gene *bcl-2* in response to treatment [70]. Favourable responses to treatment may also be facilitated by an immune response following chemotherapy. Greiner and colleagues demonstrated the immune recognition of mutated NPM1 by CD4+ and CD8+ T cells and proposed that favourable outcomes may be aided by T cell lysis of leukaemic cells following chemotherapy [73].

One of the most significant consequences of the ELN guidelines is the recommendation that patients with an *NPM1* mutation in the absence of a *FLT3* mutation that fall into the favourable risk category should not undergo allogenic haematopoietic cell transplant (HCT) at first remission. Whilst being a crucially important treatment in cases where relapse risk is expected to be high, the associated risk of potentially fatal infection due to immunosuppression and graft vs. host disease is thought to be greater than any benefit in patients with a relapse rate of less than 35% such as in NPM1mut AML falling into the favourable risk category.

## 8. Treatment Options in *NPM1*mut AML

In addition to helping categorise the risk for AML patients based on the presence or absence of mutations such as *NPM1*, it is hoped that knowledge of the mutational profile in individuals will help allow clinicians to treat these patients with therapies targeted at specific mutations present. The main treatment for AML has remained relatively unchanged for over 40 years comprising the ‘7 + 3’ chemotherapy regimen involving infusion of the cytotoxic drugs cytarabine and daunorubicin for 7 and 3 days, respectively [74]. Unsurprisingly, the lack of new treatment options has meant any increase on overall survival has been limited, with modest increases in 5-year overall survival in under 60-year-olds from 10% in the 1970s to around 40–45% in the 2000’s with complete remission occurring in 75–80% of patients following initial treatment [75,76]. The modest increase in survival is believed to be due to improved supportive care during chemotherapy treatment and improved management and support during and after stem cell transplantation. The improvement in older patients over 65 years of age has sadly not improved in a similar fashion with 5-year survival rates for over 60-year-olds approximately 17%, dropping even lower to around 5% surviving 5 years in the over 65 age group [75,77]. The disparity in survival amongst younger and older patients is partly due to older patients being unable to undergo intensive chemotherapy and the majority being ineligible for stem cell transplant leaving limited treatment options. Hypomethylating agents such as azacitidine have been commonly used in patients unsuitable for intensive treatment since the 2000’s but have resulted in limited improvements in older patients that comprise most AML patients [78]. More recently the antibody-drug combination of Gemtuzumab Ozogamicin has been added to the ‘7 + 3’ chemotherapy regimen in patients with favourable and intermediate risk, such as NPM1 mut AML [79].

What is the benefit of having molecular information at hand when it comes to treatment options? Research has shown that a high dose of daunorubicin, twice that of the standard dose, is beneficial in improving survival in patients with an *NPM1* mutation [80]. Presentation of AML is often a medical emergency, especially in cases with high white cell and/or blast count with chemotherapy being given as soon as possible. With the advent of genetic information being available to clinicians that may alter treatment, it has been queried whether delaying treatment until results of genetic testing are returned will have any effect on treatment. Conflicting research in the area has been published with one German study showing no adverse effect on survival if time from diagnosis to treatment start is delayed [81]. Whilst a study using data from a similar number of patients in the Swedish population registry found a benefit in patients treated between day 0 and 5 from diagnosis in comparison to treatment started anytime post day 5 [82]. The current uncertainty highlights the need to treat AML sufferers in as timely a fashion as possible, whilst also expediting the return of all relevant genetic testing to the clinician. In this regard, one of the most important and challenging recommendations in the ELN guidelines was that results for *NPM1* and *FLT3* mutation testing be returned within 24–48 h of diagnosis to facilitate intensive chemotherapy for all eligible patients.

Similarly, the RATIFY clinical trial, showed the advantage in returning genetic testing results in a short period of time, in this instance 48 h. RATIFY, a phase 3 trial testing the addition of the tyrosine kinase inhibitor midostaurin to the standard 7 + 3 treatment regimen in *FLT3* mutated AML showed a survival benefit in the midostaurin treated group with a hazard ratio of 0.78 in comparison to the placebo group [83].

## 9. Detection of *NPM1* Mutations in AML

With increasing knowledge of the genetic heterogeneity in AML and the specific mutations influencing the course of disease, the need for rapid detection of relevant mutations to guide clinical decision making has become crucial. Large scale, parallel DNA sequencing responsible for the discovery of many of the previously discussed genes has advanced to a level where it is economically viable and practical to be used more routinely, being available to most patients in UK during their course of treatment [84]. The creation of specific gene panels to allow testing for mutations in all relevant genes in a particular disease such as AML has become feasible through the implementation of next generation sequencing (NGS) platforms. In AML, patients should be routinely screened for up to 53 clinically relevant genes at diagnosis to aid with WHO classification and ELN risk stratification [85].

Whilst NGS is a vital tool in understanding the overall mutational landscape in individuals, it is unlikely to yet be suited in providing results in the required turnaround time of 24–48 h as recommended by the ELN for determining mutational status of *NPM1* and *FLT3*. Current guidance for return of NGS panel results from NHS England shows a turnaround time of 21 days for NGS haemato-oncology referrals [86]. It may be the case that processing fewer specimens when running NGS might help in reducing turnaround times, yet this is likely to remain prohibitively expensive in the immediate future in the clinical setting.

## 10. Rapid Testing for *NPM1* Mutations in AML

**Figure 4:**
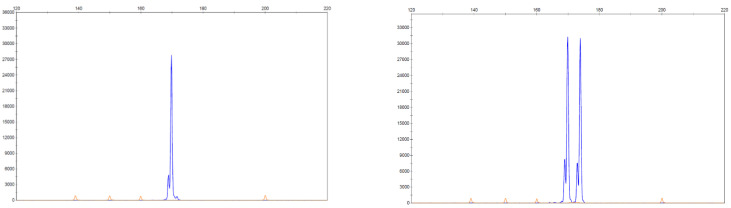
*NPM1* detection using fragment analysis by capillary electrophoresis. The image on the left shows wild type *NPM1* with the x-axis representing number of base pairs (bp) and the y-axis fluorescence. The image on the right shows the presence of a peak at 169 bp representing the wild type peak and the peak to the right at 173 bp showing the mutated *NPM1* with a common 4 bp insertion. The area underneath each curve can be used to quantify the amount of wild type and mutant NPM1 present [87].

The requirement for a rapid assay with suitable sensitivity for detecting *FLT3-ITD* mutations has led to fragment analysis by capillary electrophoresis being routinely used and this was the method of testing in the RATIFY trial. This relatively straight forward assay involves the separation of sections of a gene of choice based on size following amplification by polymerase chain reaction (PCR) and detection via fluorescence. This assay is suited to detecting *FLT-ITD* mutations as the mutation results in a fragment size larger than the wild type *FLT3* gene. Similarly, it has been suggested that this method would be ideal in testing for *NPM1* mutations as approximately 95% of mutations result in a 4-base pair insertion that can be separated from the wild type using capillary electrophoresis giving a rapid, qualitative result with high sensitivity, being able to detect down to 2% mutant allele [87]. Figure 4 shows an example of an expected result using capillary electrophoresis to detect an *NPM1* mutation.

Another benefit in using fragment analysis is in determining the mutational burden, essentially the proportion of mutant alleles in comparison to the wild type of the gene in question. This is commonly done by calculating the allelic ratio (AR) of the mutant present by dividing the area under the curve of the mutant allele fragment by the area under the wild type allele [88]. Allelic ratios >0.5 are classified as a high ratio and <0.5 as being low, with these definitions being included and contributing to prognosis in the ELN guidelines with a higher AR being associated with a poorer prognosis in *FLT3-ITD* mutated AML.

Whilst AR is commonly used in line with international guidance in *FLT3* mutation reporting, the benefits of reporting mutational burden in NPM1mut cases has yet to be fully demonstrated and is currently debated, with some research indicating that an increased mutational burden in NPM1mut AML leads to a significant reduction in overall and event free survival [89]. This claim has been disputed with it being argued that once adjusted for known, adverse confounding factors such as high initial leukocyte and blast counts as well as unfavourable co-mutations, a high mutational burden did not independently predict poorer survival [90]. The utility of mutational burden in *NPM1* mutated AML is still being debated and may arguably be of importance in rapid diagnostic testing as knowledge improves.

In assessing disease status of patients through their course of treatment, NPM1mut also provides a useful target for minimal residual disease (MRD) monitoring. As *NPM1* mutations do not drive clonal haematopoiesis and are highly expressed, the presence of detectable levels of NPM1mut transcripts at various points in treatment can be determined using a highly sensitive assay such as real time quantitative PCR (RT-qPCR) or droplet digital PCR (dPCR) [91,92]. The continued presence of NPM1mut transcripts has been shown to be detectable in 15% of patients following second round chemotherapy and is associated with an 82 % chance of relapse versus 30% in patients with non-detectable NPM1-mutated transcripts [93]. MRD status prior to allogenic stem cell transplant (HSCT) has also been shown to be a good predictor of outcome with recent research showing MRD negative patients having a 2-year overall survival of 83% versus 13% in a group that had high MRD [94]. The risk of low positivity MRD patients was dependent on the associated *FLT3-ITD* status with *FLT3-ITD* positive patients shown to be high risk and *FLT3-ITD* negative patients shown to be low risk, respectively. Conversely, the detection of NPM mut MRD prior to HSCT is associated with inferior survival [94]. Having a sensitive assay for MRD monitoring of *NPM1* in AML will have to be a consideration for laboratories in large testing centres with standardisation and access to external quality assessment programmes being required.

## 11. Future Developments in *NPM1*mut AML Treatment

The multiple, diverse functions of *NPM1* have provided researchers with challenges and opportunities. Challenges in the numerous ways NPM1mut may contribute to the development of AML that are still not fully understood and opportunities in the multiple therapeutic targets that exist and are being actively investigated.

Some therapeutic targets include inhibitors of the nuclear exporter XPO1 such as Selinexor with the aim of correcting the aberrant cytoplasmic localisation of NPM1mut [95]. The significance of *HOX* gene expression has proven to be another target with inhibitors of MLL1-menin shown to have anti-leukaemic activity in *NPM1* mutated leukaemia [56]. There is evidence that NPM1mut cells may be more susceptible to eradication by inducing nucleolar stress, with a long available RNA polymerase I inhibitor dactinomycin being shown to be effective in leading to remission in NPM1mut AML [96]. As previously discussed, the ability of specific T cell recognition has led researchers to investigate the potential of using immunotherapy targeted at *NPM1* mutated AML with recent research in mice showing the possibility of using chimeric antigen receptor T cell (CAR-T) therapy in the future [97].

One of the most promising treatments for older individuals is in the use of the bcl-2 inhibitor Venetoclax, commonly used in B-cell malignancies [98]. Recent research has shown it to be highly effective in treating NPM1mut AML in combination with hypomethylating agents with patients over 65 years old showing a 69% reduction in mortality in comparison with those treated with intensive chemotherapy alone [99]. A UK led, international clinical trial VICTOR (Venetoclax or intensive chemotherapy for treatment of favourable risk AML) investigating the efficacy of Venetoclax versus standard intense chemotherapy in over 60 year olds with NPM1mut AML is currently underway [100]. Such is the promise of Venetoclax treatment that emergency approval in the UK was granted for AML patients as an additional treatment option during the SARS-CoV-2 pandemic by the national institute for health and care excellence (NICE) with a decision on its full approval by NICE expected in November 2021 [101,102]. It is therefore conceivable that with emerging specific agents, treatments may become more personalised with specific agents being utilized in response to specific mutations.

With rapidly expanding molecular diagnostic techniques and new technological methods, further insights into AML are likely to be elucidated. Recent research using such techniques as RNA sequencing and chromatin accessibility analysis suggests there are two distinct NPM1mut subtypes classified as primitive and committed, differing in their expression profiles [103]. The primitive subtype was shown to have worse overall survival, yet also higher sensitivity in ex vivo drug sensitivity profiling to several kinase inhibitors including Sorafenib, Sunitinib and Ruxolitinib when compared with the committed subtype, regardless of *FLT3-ITD* status. Such research highlights that there are likely to be further discoveries in this area in the near future and that laboratories may have to adapt at relatively short notice to facilitate such testing.

## 12. Summary

Rapidly expanding knowledge of the role of genetic mutations such as *NPM1* in the development of such an aggressive and difficult to treat disease like AML has led to more accurate prognoses and several potential treatment options where there has been few before, particularly for elderly patients. The speed of change gives rise to difficulties in translating such knowledge to the clinical area with laboratories having to be ever more adept at providing fast, reliable results to help guide clinical decisions. With rapid advancements and potential therapeutic options being developed, the utility in providing clinicians with *NPM1* and *FLT3* status in a matter of days as opposed to weeks is evident, with the hope that such results can guide early decisions regarding treatment and help patients receive the most appropriate, personalised treatment at the most appropriate time which will hopefully lead to improved outcomes.

## Figures and Tables

**Table 1:** The 2016 revision of World Health Organisation classification of acute myeloid leukaemia (AML) and related neoplasms [14].

Acute Myeloid Leukemia (AML) and Related Neoplasms
AML with recurrent genetic abnormalities
AML with t(8;21)(q22;q22.1); *RUNX1-RUNX1T1*
AML with inv(16)(p13.1q22) or t(16;16)(p13.1;q22); *CBFB-MYH11*
APL with *PML-RARA* t(15;17)
AML with t(9;11)(p21.3;q23.3); *MLLT3-KMT2A*
AML with t(6;9)(p23;q34.1); *DEK-NUP214*
AML with inv(3)(q21.3q26.2) or t(3;3)(q21.3;q26.2); *GATA2*, *MECOM*
AML (megakaryoblastic) with t(1;22)(p13.3;q13.3); *RBM15-MKL1*
Provisional entity: AML with *BCR-ABL1*
AML with mutated *NPM1*
AML with biallelic mutations of *CEBPA*
Provisional entity: AML with mutated *RUNX1*

**Table 2:** The 2017 European Leukaemia Network risk stratification of AML [69].

Risk Category	Genetic Abnormality
Favorable	t(8;21)(q22;q22.1); * RUNX1-RUNX1T1 *
	inv(16)(p13.1q22) or t(16;16)(p13.1;q22); * CBFB-MYH11 *
	Mutated *NPM1* without *FLT3*-ITD or with *FLT3*-ITD^low^
	Biallelic mutated * CEBPA *
Intermediate	Mutated *NPM1* and *FLT3*-ITD^high^
	Wild-type *NPM1* without *FLT3*-ITD or with *FLT3*-ITD^low^ (without adverse-risk genetic lesions)
	t(9;11)(p21.3;q23.3); * MLLT3-KMT2A *
	Cytogenetic abnormalities not classified as favorable or adverse
Adverse	t(6;9)(p23;q34.1); * DEK * - * NUP214 *
	t(v;11q23.3); * KMT2A * rearranged
	t(9;22)(q34.1;q11.2); * BCR * - * ABL1 *
	inv(3)(q21.3q26.2) or t(3;3)(q21.3;q26.2); * GATA2 * , * MECOM(EVI1) *
	−5 or del(5q); −7; −17/abn(17p)
	Complex karyotype, monosomal karyotype
	Wild-type *NPM1* and *FLT3*-ITD^high^
	Mutated * RUNX1 *
	Mutated * ASXL1 *
	Mutated * TP53 *

## Data Availability

Not applicable.

## References

[1] Roman E., Smith A., Appleton S., Crouch S., Kelly R., Kinsey S., Cargo C., Patmore R. (2016). Myeloid malignancies in the real-world: Occurrence, progression and survival in the UK’s population-based haematological malignancy research network 2004–15. Cancer Epidemiol..

[2] Cancer Research UK Acute Myeloid Leukaemia (AML) Incidence Statistics. https://www.cancerresearchuk.org/health-professional/cancer-statistics/statistics-by-cancer-type/leukaemia-aml/incidence#heading-Zero.

[3] Cancer Research UK Acute Myeloid Leukaemia (AML) Mortality Statistics. https://www.cancerresearchuk.org/health-professional/cancer-statistics/statistics-by-cancer-type/leukaemia-aml/mortality.

[4] Bennett J.H. (1845). Case of hypertrophy of the spleen and liver, in which death took place from suppuration of the blood. Edinb. Med Surg. J..

[5] Virchow R. (1845). Weisses blut. Froriep’s Not..

[6] Virchow R. (1847). Zur pathologischen physiologie des blutes. II. Weisses blut. Arch. Pathol. Anat. Physiol..

[7] Ehrlich P. (1877). Beitrag zur kenntnis der anilinfarbungen under ihrer verwendung in der microskopischen technik. Arch. Mikrochir. Anat..

[8] Reschad H., Schilinng-Torgau V. (1913). Ueber eine neue leukamie dursh echte uebergangsformen und ihre bedeutung fur dies selbstãndigkeit diezer zellen. Munch. Med. Wochenschr..

[9] Bennett J.M., Catovsky D., Daniel M.-T., Flandrin G., Galton D.A.G., Gralnick H.R., Sultan C. (1976). Proposals for the classification of the acute leukaemias French-American-British (FAB) co-operative group. Br. J. Haematol..

[10] Sanger F., Nicklen S., Coulson A.R. (1977). DNA sequencing with chain-terminating inhibitors. Proc. Natl. Acad. Sci. USA.

[11] Rudkin G.T., Stollar B.D. (1977). High resolution detection of DNA–RNA hybrids in situ by indirect immunofluorescence. Nature.

[12] Jaffe E.S., Harris N.L., Stein H., Vardiman J.W., Jaffe E.S., Harris N.L., Stein H., Vardiman J.W. (2001). Pathology and Genetics of Tumours of Haematopoietic and Lymphoid Tissues.

[13] Campo E., Harris N.L., Jaffe E.S., Pileri S.A., Stein H., Thiele J., Vardiman J.W. (2008). WHO Classifycation of Tumours of Haematopoietic and Lymphoid Tissues.

[14] Arber D.A., Orazi A., Hasserjian R., Thiele J., Borowitz M.J., Le Beau M.M., Bloomfield C.D., Cazzola M., Vardiman J.W. (2016). The 2016 revision to the World Health Organization classification of myeloid neoplasms and acute leukemia. Blood.

[15] Doorn-Khosrovani S.B.V.W.V., Erpelinck C., Meijer J., Van Oosterhoud S., Van Putten W.L.J., Valk P.J.M., Beverloo H.B., Tenen D., Löwenberg B., Delwel R. (2003). Biallelic mutations in the CEBPA gene and low CEBPA expression levels as prognostic markers in intermediate-risk AML. Hematol. J..

[16] Box J.K., Paquet N., Adams M.N., Boucher D., Bolderson E., O’Byrne K.J., Richard D.J. (2016). Nucleophosmin: From structure and function to disease development. BMC Mol. Biol..

[17] Zarka J., Short N.J., Kanagal-Shamanna R., Issa G.C. (2020). Nucleophosmin 1 mutations in acute myeloid leukemia. Genes.

[18] Kojima K., Kornblau S.M., Ruvolo V., Dilip A., Duvvuri S., Davis R.E., Zhang M., Wang Z., Coombes K.R., Zhang N. (2013). Prognostic impact and targeting of CRM1 in acute myeloid leukemia. Blood.

[19] Mitrea D.M., Grace C.R., Buljan M., Yun M.-K., Pytel N.J., Satumba J., Nourse A., Park C.-G., Babu M.M., White S.W. (2014). Structural polymorphism in the N-terminal oligomerization domain of NPM1. Proc. Natl. Acad. Sci. USA.

[20] Falini B., Brunetti L., Sportoletti P., Martelli M.P. (2020). NPM1-mutated acute myeloid leukemia: From bench to bedside. Blood.

[21] Yu Y., Maggi L.B., Brady S.N., Apicelli A.J., Dai M.-S., Lu H., Weber J.D. (2006). Nucleophosmin is essential for ribosomal protein L5 nuclear export. Mol. Cell. Biol..

[22] Cela I., Di Matteo A., Federici L. (2020). Nucleophosmin in its interaction with ligands. Int. J. Mol. Sci..

[23] Gadad S., Senapati P., Syed S.H., Rajan R.E., Shandilya J., Swaminathan V., Chatterjee S., Colombo E., Dimitrov S., Pelicci P.G. (2011). The multifunctional protein nucleophosmin (NPM1) is a human linker histone H1 chaperone. Biochemistry.

[24] Swaminathan V., Kishore A.H., Febitha K.K., Kundu T.K. (2005). Human histone chaperone nucleophosmin enhances acetylation-dependent chromatin transcription. Mol. Cell. Biol..

[25] Wang W., Budhu A., Forgues M., Wang X.W. (2005). Temporal and spatial control of nucleophosmin by the Ran–Crm1 complex in centrosome duplication. Nat. Cell Biol..

[26] Okuda M., Horn H., Tarapore P., Tokuyama Y., Smulian A., Chan P.-K., Knudsen E.S., Hofmann I.A., Snyder J.D., Bove K.E. (2000). Nucleophosmin/B23 is a target of CDK2/Cyclin E in centrosome duplication. Cell.

[27] Grisendi S., Bernardi R., Rossi M., Cheng K., Khandker L., Manova K., Pandolfi P.P. (2005). Role of nucleophosmin in embryonic development and tumorigenesis. Nat. Cell Biol..

[28] Scheer U., Hock R. (1999). Structure and function of the nucleolus. Curr. Opin. Cell Biol..

[29] Feric M., Vaidya N., Harmon T.S., Mitrea D.M., Zhu L., Richardson T.M., Kriwacki R.W., Pappu R.V., Brangwynne C.P. (2016). Coexisting liquid phases underlie nucleolar subcompartments. Cell.

[30] Mitrea D.M., Cika J.A., Stanley C.B., Nourse A., Onuchic P., Banerjee P.R., Phillips A.H., Park C.-G., Deniz A.A., Kriwacki R.W. (2018). Self-interaction of NPM1 modulates multiple mechanisms of liquid–liquid phase separation. Nat. Commun..

[31] Poletto M., Malfatti M.C., Dorjsuren D., Scognamiglio P.L., Marasco D., Vascotto C., Jadhav A., Maloney D.J., Wilson D.M., Simeonov A. (2016). Inhibitors of the apurinic/apyrimidinic endonuclease 1 (APE1)/nucleophosmin (NPM1) interaction that display anti-tumor properties. Mol. Carcinog..

[32] Kurki S., Peltonen K.D., Latonen L., Kiviharju T.M., Ojala P., Meek D., Laiho M. (2004). Nucleolar protein NPM interacts with HDM2 and protects tumor suppressor protein p53 from HDM2-mediated degradation. Cancer Cell.

[33] Grisendi S., Mecucci C., Falini B., Pandolfi P.P. (2006). Nucleophosmin and cancer. Nat. Rev. Cancer.

[34] Döhner H., Weisdorf D.J., Bloomfield C.D. (2015). Acute myeloid leukemia. N. Engl. J. Med..

[35] Braoudaki M., Papathanassiou C., Katsibardi K., Tourkadoni N., Karamolegou K., Tzortzatou-Stathopoulou F. (2010). The frequency of NPM1 mutations in childhood acute myeloid leukemia. J. Hematol. Oncol..

[36] Suzuki T., Kiyoi H., Ozeki K., Tomita A., Yamaji S., Suzuki R., Kodera Y., Miyawaki S., Asou N., Kuriyama K. (2005). Clinical characteristics and prognostic implications of NPM1 mutations in acute myeloid leukemia. Blood.

[37] Bain B.J., Heller M., Toma S., Pavlu J. (2015). The cytological features of NPM1 -mutated acute myeloid leukemia. Am. J. Hematol..

[38] Bain B.J., Béné M.C. (2019). Morphological and immunophenotypic clues to the WHO categories of acute myeloid leukaemia. Acta Haematol..

[39] Redner R., Rush E., Faas S., Rudert W., Corey S. (1996). The t(5;17) variant of acute promyelocytic leukemia expresses a nucleophosmin-retinoic acid receptor fusion. Blood.

[40] Morris S.W., Kirstein M.N., Valentine M.B., Dittmer K.G., Shapiro D.N., Saltman D.L., Look A.T. (1994). Fusion of a kinase gene ALK, to a nucleolar protein gene, NPM, in non-Hodgkin’s lymphoma. Science.

[41] Raimondi S.C., Dubé I.D., Valentine M.B., Mirro J., Watt H.J., Larson R., Bitter M.A., Le Beau M.M., Rowley J.D. (1989). Clinicopathologic manifestations and breakpoints of the t(3;5) in patients with acute nonlymphocytic leukemia. Leukemia.

[42] Martelli M.P., Rossi R., Venanzi A., Meggendorfer M., Perriello V.M., Martino G., Spinelli O., Ciurnelli R., Varasano E., Brunetti L. (2021). Novel NPM1 exon 5 mutations and gene fusions leading to aberrant cytoplasmic nucleophosmin in AML. Blood.

[43] Gu T.-L., Tothova Z., Scheijen B., Griffin J.D., Gilliland D.G., Sternberg D.W. (2004). NPM-ALK fusion kinase of anaplastic large-cell lymphoma regulates survival and proliferative signaling through modulation of FOXO3a. Blood.

[44] Falini B., Nicoletti I., Martelli M.F., Mecucci C. (2006). Acute myeloid leukemia carrying cytoplasmic/mutated nucleophosmin (NPMc+ AML): Biologic and clinical features. Blood.

[45] Falini B., Mecucci C., Tiacci E., Alcalay M., Rosati R., Pasqualucci L., La Starza R., Diverio D., Colombo E., Santucci A. (2005). Cytoplasmic nucleophosmin in acute myelogenous leukemia with a normal karyotype. N. Engl. J. Med..

[46] Kunchala P., Kuravi S., Jensen R., McGuirk J., Balusu R. (2018). When the good go bad: Mutant NPM1 in acute myeloid leukemia. Blood Rev..

[47] Borrow J., Dyer S.A., Akiki S., Griffiths M.J. (2019). Molecular roulette: Nucleophosmin mutations in AML are orchestrated through N-nucleotide addition by TdT. Blood.

[48] Repasky J.A.E., Corbett E., Boboila C., Schatz D.G. (2004). Mutational analysis of terminal deoxynucleotidyltransferase-mediated N-nucleotide addition in V(D)J recombination. J. Immunol..

[49] Motea E.A., Berdis A.J. (2010). Terminal deoxynucleotidyl transferase: The story of a misguided DNA polymerase. Biochim. Biophys. Acta (BBA) Proteins Proteom..

[50] Bolli N., Nicoletti I., De Marco M.F., Bigerna B., Pucciarini A., Mannucci R., Martelli M.P., Liso A., Mecucci C., Fabbiano F. (2007). Born to be exported: COOH-terminal nuclear export signals of different strength ensure cytoplasmic accumulation of nucleophosmin leukemic mutants. Cancer Res..

[51] Arregi I., Falces J., Olazabal-Herrero A., Alonso-Mariño M., Taneva S.G., Rodriguez J.A., Urbaneja M.A., Bañuelos S. (2015). Leukemia-associated mutations in nucleophosmin alter recognition by CRM1: Molecular basis of aberrant transport. PLoS ONE.

[52] Braekeleer E.D., Douet-Guilbert N., Basinko A., Bris M.J.L., Morel F., Braekeleer M.D. (2014). Hox gene dysregulation in acute myeloid leukemia. Future Oncol..

[53] Spencer D.H., Young M.A., Lamprecht T.L., Helton N.M., Fulton R., O’Laughlin M., Fronick C., Magrini V., Demeter R.T., Miller C.A. (2015). Epigenomic analysis of the HOX gene loci reveals mechanisms that may control canonical expression patterns in AML and normal hematopoietic cells. Leukemia.

[54] Brunetti L., Gundry M.C., Sorcini D., Guzman A.G., Huang Y.-H., Ramabadran R., Gionfriddo I., Mezzasoma F., Milano F., Nabet B. (2018). Mutant NPM1 Maintains the leukemic state through HOX expression. Cancer Cell.

[55] Gundry M.C., Goodell M.A., Brunetti L. (2020). It’s all about meis: Menin-mll inhibition eradicates NPM1-mutated and mll-rearranged acute leukemias in mice. Cancer Cell.

[56] Kühn M.W.M., Song E., Feng Z., Sinha A., Chen C.-W., Deshpande A.J., Cusan M., Farnoud N., Mupo A., Grove C. (2016). Targeting chromatin regulators inhibits leukemogenic gene expression in NPM1 mutant leukemia. Cancer Discov..

[57] Gu X., Ebrahem Q., Mahfouz R.Z., Hasipek M., Enane F., Radivoyevitch T., Rapin N., Przychodzen B., Hu Z., Balusu R. (2018). Leukemogenic nucleophosmin mutation disrupts the transcription factor hub that regulates granulomonocytic fates. J. Clin. Investig..

[58] Pianigiani G., Betti C., Bigerna B., Rossi R., Brunetti L. (2020). PU.1 subcellular localization in acute myeloid leukaemia with mutated NPM1. Br. J. Haematol..

[59] Gu X., Saunthararajah Y. (2020). Cytoplasmic dislocation of NPM1 and PU.1 in NPM1 -mutated leukaemia is obscured by paraformaldehyde fixation. Br. J. Haematol..

[60] Dawson M.A., Gudgin E., Horton S.J., Giotopoulos G., Meduri E., Robson S., Cannizzaro E., Osaki H., Wiese M.D., Putwain S. (2014). Recurrent mutations, including NPM1c, activate a BRD4-dependent core transcriptional program in acute myeloid leukemia. Leukemia.

[61] Hartmann L., Metzeler K.H. (2019). Clonal hematopoiesis and preleukemia—Genetics, biology, and clinical implications. Genes Chromosom Cancer.

[62] Park D.J., Kwon A., Cho B.S., Kim H.J., Hwang K.A., Kim M., Kim Y. (2020). Characteristics of DNMT3A mutations in acute myeloid leukemia. Blood Res..

[63] Mason E.F., Hasserjian R.P., Aggarwal N., Seegmiller A.C., Pozdnyakova O. (2019). Blast phenotype and comutations in acute myeloid leukemia with mutated NPM1 influence disease biology and outcome. Blood Adv..

[64] Corces M., Hong W.-J., Weissman I.L., Medeiros B.C., Majeti R. (2014). Preleukemic mutations in human acute myeloid leukemia affect epigenetic regulators and persist in remission. Proc. Natl. Acad. Sci. USA.

[65] Daver N., Schlenk R.F., Russell N.H., Levis M.J. (2019). Targeting FLT3 mutations in AML: Review of current knowledge and evidence. Leukemia.

[66] Arceci R.J., Berman J.N., Meshinchi S., Dellaire G., Berman J.N., Arceci R.J. (2014). Acute Myeloid Leukemia in Cancer Genomics.

[67] Papaemmanuil E., Gerstung M., Bullinger L., Gaidzik V.I., Paschka P., Roberts N.D., Potter N.E., Heuser M., Thol F., Bolli N. (2016). Genomic classification and prognosis in acute myeloid leukemia. N. Engl. J. Med..

[68] Guryanova O.A., Shank K., Spitzer B., Luciani L., Koche R.P., Garrett-Bakelman F.E., Ganzel C., Durham B.H., Mohanty A., Hoermann G. (2016). DNMT3A mutations promote anthracycline resistance in acute myeloid leukemia via impaired nucleosome remodeling. Nat. Med..

[69] Döhner H., Estey E., Grimwade D., Amadori S., Appelbaum F.R., Büchner T., Dombret H., Ebert B.L., Fenaux P., Larson R.A. (2017). Diagnosis and management of AML in adults: 2017 ELN recommendations from an international expert panel. Blood.

[70] Zhang S., Qin F., Yang L., Xian J., Zou Q., Jin H., Wang L., Zhang L. (2016). Nucleophosmin mutations induce chemosensitivity in THP-1 leukemia cells by suppressing NF-κB activity and regulating Bax/Bcl-2 expression. J. Cancer.

[71] Guzman M.L., Neering S.J., Upchurch D., Grimes B., Howard D.S., Rizzieri D.A., Luger S.M., Jordan C.T. (2001). Nuclear factor-κB is constitutively activated in primitive human acute myelogenous leukemia cells. Blood.

[72] Bosman M.C.J., Schuringa J.J., Vellenga E. (2016). Constitutive NF-κB activation in AML: Causes and treatment strategies. Crit. Rev. Oncol. Hematol..

[73] Greiner J., Schneider V., Schmitt M., Götz M., Döhner K., Wiesneth M., Döhner H., Hofmann S. (2013). Immune responses against the mutated region of cytoplasmatic NPM1 might contribute to the favorable clinical outcome of AML patients with NPM1 mutations (NPM1mut). Blood.

[74] Dombret H., Gardin C. (2016). An update of current treatments for adult acute myeloid leukemia. Blood.

[75] Rowe J.M. (2019). Will new agents impact survival in AML?. Best Pract. Res. Clin. Haematol..

[76] Burnett A.K., Hills R.K., Milligan D., Kjeldsen L., Kell J., Russell N.H., Yin J.A., Hunter A., Goldstone A.H., Wheatley K. (2011). Identification of patients with acute myeloblastic leukemia who benefit from the addition of gemtuzumab ozogamicin: Results of the MRC AML15 Trial. J. Clin. Oncol..

[77] Cancer Research UK Acute Myeloid Leukaemia (AML) Survival Statistics. https://www.cancerresearchuk.org/about-cancer/acute-myeloid-leukaemia-aml/survival.

[78] Zeidan A.M., Wang R., Wang X., Shallis R.M., Podoltsev N.A., Bewersdorf J.P., Huntington S.F., Neparidze N., Giri S., Gore S.D. (2020). Clinical outcomes of older patients with AML receiving hypomethylating agents: A large population-based study in the United States. Blood Adv..

[79] Heuser M., Ofran Y., Boissel N., Brunet Mauri S., Craddock C., Janssen J., Wierzbowska A., Buske C. (2020). Acute myeloid leukaemia in adult patients: ESMO clinical practice guidelines for diagnosis, treatment and follow-up. Ann. Oncol..

[80] Luskin M.R., Lee J.-W., Fernandez H.F., Abdel-Wahab O., Bennett J.M., Ketterling R.P., Lazarus H.M., Levine R.L., Litzow M.R., Paietta E.M. (2016). Benefit of high-dose daunorubicin in AML induction extends across cytogenetic and molecular groups. Blood.

[81] Röllig C., Kramer M., Schliemann C., Mikesch J.-H., Steffen B., Krämer A., Noppeney R., Schäfer-Eckart K., Krause S.W., Haenel M. (2020). Does time from diagnosis to treatment affect the prognosis of patients with newly diagnosed acute myeloid leukemia?. Blood.

[82] Juliusson G., Hagberg O., Lazarevic V.L., Lehmann S., Höglund M. (2021). Impact of treatment delay in acute myeloid leukemia revisited. Blood Adv..

[83] Stone R.M., Mandrekar S.J., Sanford B.L., Laumann K., Geyer S., Bloomfield C.D., Thiede C., Prior T.W., Döhner K., Marcucci G. (2017). Midostaurin plus chemotherapy for acute myeloid leukemia with aFLT3Mutation. N. Engl. J. Med..

[84] Mardis E.R., Ding L., Dooling D.J., Larson D.E., McLellan M.D., Chen K., Koboldt D.C., Fulton R.S., Delehaunty K.D., McGrath S.D. (2009). Recurring mutations found by sequencing an acute myeloid leukemia genome. N. Engl. J. Med..

[85] Aguilera-Diaz A., Vazquez I., Ariceta B., Mañú A., Blasco-Iturri Z., Palomino-Echeverría S., Larrayoz M.J., Garcia-Sanz R., Prieto-Conde M.I., Chillón M.D.C. (2020). Assessment of the clinical utility of four NGS panels in myeloid malignancies. Suggestions for NGS panel choice or design. PLoS ONE.

[86] Wessex Regional Genetics Laboratory NHS England Target Report Turnaround Times. https://www.salisbury.nhs.uk/media/5teba2sh/websitetatsdocumentoctober2018.pdf.

[87] Behdad A., Betz B. (2017). Molecular testing in acute myeloid leukemia. Diagnostic Molecular Pathology.

[88] Yalniz F., Abou Dalle I., Kantarjian H., Borthakur G., Kadia T., Patel K., Loghavi S., Garcia-Manero G., Sasaki K., Daver N. (2019). Prognostic significance of baseline FLT3-ITD mutant allele level in acute myeloid leukemia treated with intensive chemotherapy with/without sorafenib. Am. J. Hematol..

[89] Patel S.S., Kuo F.C., Gibson C.J., Steensma D.P., Soiffer R.J., Alyea III E.P., Chen Y.B.A., Fathi A.T., Graubert T.A., Brunner A.M. (2018). High NPM1-mutant allele burden at diagnosis predicts unfavorable outcomes in de novo AML. Blood.

[90] Rothenberg-Thurley M., Herold T., Görlich D., Sauerland C., Janke H., Prassek V.V., Konstandin N.P., Dufour A.M., Schneider S., Ksienzyk B. (2018). NPM1 variant allele frequency and outcomes in AML. Blood.

[91] Falini B., Sciabolacci S., Falini L., Brunetti L., Martelli M.P. (2021). Diagnostic and therapeutic pitfalls in NPM1-mutated AML: Notes from the field. Leukemia.

[92] Trinchant N.M., Hu Y., Alas M.A., Ali F., Wouters B.J., Lee S., Ritchie E.K., Desai P., Guzman M.L., Roboz G.J. (2017). Minimal residual disease monitoring of acute myeloid leukemia by massively multiplex digital PCR in patients with NPM1 mutations. J. Mol. Diagn..

[93] Ivey A., Hills R., Simpson M., Jovanovic J.V., Gilkes A., Grech A., Patel Y., Bhudia N., Farah H., Mason J. (2016). Assessment of minimal residual disease in standard-risk AML. N. Engl. J. Med..

[94] Dillon R., Hills R., Freeman S., Potter N., Jovanovic J., Ivey A., Kanda A.S., Runglall M., Foot N., Valganon M. (2020). Molecular MRD status and outcome after transplantation in NPM1-mutated AML. Blood.

[95] Garzon R., Savona M., Baz R., Andreeff M., Gabrail N., Gutierrez M., Savoie L., Mau-Sørensen M., Wagner-Johnston N., Yee K. (2017). A phase 1 clinical trial of single-agent selinexor in acute myeloid leukemia. Blood.

[96] Falini B., Brunetti L., Martelli M.P. (2015). Dactinomycin in NPM1-mutated acute myeloid leukemia. N. Engl. J. Med..

[97] Xie G., Ivica N.A., Jia B., Li Y., Dong H., Liang Y., Brown D., Romee R., Chen J. (2020). CAR-T cells targeting a nucleophosmin neoepitope exhibit potent specific activity in mouse models of acute myeloid leukaemia. Nat. Biomed. Eng..

[98] Scheffold A., Jebaraj B.M.C., Stilgenbauer S. (2018). Venetoclax: Targeting BCL2 in hematological cancers. Small Mol. Hematol..

[99] Lachowiez C.A., Loghavi S., Kadia T.M., Daver N., Borthakur G., Pemmaraju N., Naqvi K., Alvarado Y., Yilmaz M., Short N. (2020). Outcomes of older patients with NPM1-mutated AML: Current treatments and the promise of venetoclax-based regimens. Blood Adv..

[100] King’s College London Study Investigating Treatment for Acute Myeloid Leukaemia Launches at King’s. https://www.kcl.ac.uk/news/study-investigating-treatment-for-acute-myeloid-leukaemia-announced-at-kings.

[101] National Institute for Health and Care Excellence NHS England Interim Treatment Options. https://www.nice.org.uk/guidance/ng161/resources/interim-treatment-change-options-during-the-covid19-pandemic-endorsed-by-nhs-england-pdf-8715724381.

[102] National Institute for Health and Care Excellence Venetoclax with a Hypomethylating Agent or Low Dose Cytarabine for Untreated Acute Myeloid Leukaemia when Intensive Chemotherapy Is Unsuitable. https://www.nice.org.uk/guidance/indevelopment/gid-ta10478.

[103] Mer A.S., Heath E.M., Tonekaboni S.A.M., Dogan-Artun N., Nair S.K., Murison A., Garcia-Prat L., Shlush L., Hurren R., Voisin V. (2021). Biological and therapeutic implications of a unique subtype of NPM1 mutated AML. Nat. Commun..

